# Evaluation of clinical outcome and predictive factors for thromboembolism or hemorrhagic complications in patients treated for chronic subdural hematoma. A prospective observational study

**DOI:** 10.1007/s10143-025-03441-0

**Published:** 2025-03-17

**Authors:** Alba Scerrati, Giorgio Mantovani, Michele Alessandro Cavallo, Maria Elena Flacco, Pietro Zangrossi, Silvia Eichner, Luca Ricciardi, Antonella Mangraviti, Antonino Raco, Tamara Ius, Daniele Piccolo, Oriela Rustemi, Fabio Raneri, Carmelo Lucio Sturiale, Alberto Benato, Giovanni Pennisi, Francesco Signorelli, Gianluca Trevisi, Donato Carlo Zotta, Giorgio Lofrese, Lorenzo Mongardi, Paul Roblot, Nicola Montemurro, Rosario Maugeri, Mariachiara Sensi, Pasquale De Bonis

**Affiliations:** 1 Department of Translational Medicine, University of Ferrara, Ferrara, Italy; 2 Department of Neurosurgery, Sant’Anna University Hospital of Ferrara, Ferrara, Italy; 3 Environmental and Preventive Sciences, University of Ferrara, Ferrara, Italy; 4 Department of Neuroscience, Neurosurgical Unit, Mental Health, and Sensory Organs, Sapienza University of Rome, Rome, Italy; 5 Head-Neck and Neurosciences Department, Neurosurgery Unit, Santa Maria Della Misericordia University Hospital, Udine, Italy; 6 Department of Neurosurgery, ULSS8 Berica, Vicenza, Italy; 7 Department of Neurosurgery, Fondazione Policlinico Universitario A. Gemelli IRCCS, Università Cattolica del Sacro Cuore, Largo Agostino Gemelli, 8, Rome, RM 00136 Italy; 8 Department of Neurosciences, Imaging, and Clinical Sciences, G.D’Annunzio University, Neurosurgical Unit, Ospedale Spirito Santo, Chieti-Pescara, Italy; 9 Neurosurgery Unit, Bufalini Hospital, Cesena, Italy; 10 Service de Neurochirurgie, Hôpital Pellegrin, CHU de Bordeaux, Pl. Amélie Raba Léon, Bordeaux, France; 11 Department of Neurosurgery, Azienda Ospedaliero Universitaria Pisana, University of Pisa, Pisa, Italy; 12 Department of Biomedicine, Neurosciences and Advanced Diagnostics, School of Medicine, Neurosurgery Unit, University of Palermo, Palermo, Italy; 13 Neurology Department, Sant’Anna University Hospital of Ferrara, Ferrara, Italy

**Keywords:** Chronic subdural hematoma, Anticoagulant, Antiplatelet, CHA₂DS₂-VASc, HAS-BLED, Hemorrhagic complications, Thromboembolic complications

## Abstract

The impact of anticoagulant and antiplatelet medications on clinical outcome and risk of complications is uncertain in chronic subdural hematoma (CSDH) patients. Currently, evidence-based guidelines and specific neurosurgical scores lacks. CHA₂DS₂-VASc and HAS-BLED scores have been proven to help predicting complications in the perioperative period of non-cardiac surgeries. We performed a multicenter prospective observational trial to evaluate the clinical outcomes and complications of CSDH patient undergoing surgery, comparing patients taking anticoagulant/antiplatelet (AAPT) and not (NT). Additionally, we investigated the role of CHA₂DS₂-VASc and HAS-BLED scores in predicting thromboembolic events or hemorrhages. No associations have been found between AAPT and clinical outcomes of patients. Emergency surgery was not a significant factor in improving outcomes. Post-operative hemorrhages were more frequent in the AAPT group, but none required a second surgery. A significant higher risk of of new bleedings was found in the ASA group with discontinuation ≤ 5 days. A higher HAS-BLED score was not associated with a worse clinical outcome. A 1-point increase in CHA₂DS₂-VASc was associated with a lower probability of favorable outcomes at 1 month. 90% of AAPT and 44% of NT patients were at moderate-high risk of thromboembolic events based on CHA₂DS₂-VASc score, with no difference in incidence between groups. The use of AAPT does not influence outcomes, complication rates, or recurrence in patients undergoing surgery for CSDH. Scores such as CHA₂DS₂-VASc or HAS-BLED could aid in stratifying bleeding and thromboembolic risks and in the management of these drugs in the perioperative period.

## Introduction

Chronic subdural hematoma (CSDH) is one of the most common neurosurgical conditions, predominantly affecting the elderly population. The use of anticoagulant (AC) or antiplatelet (AP) medications has been shown to increase the risk of developing a CSDH [5, 8]. However, several studies have shown that these drugs do not influence the outcome or the risk of complications, such as rebleeding or recurrence, after surgical treatment [1, 7, 17].

Currently, there are no clear evidence-based guidelines or specific neurosurgical risk scores for managing antiplatelets or anticoagulants in patients with CSDH. This lack of guidelines leads neurosurgeons to manage the interruption or resumption of these drugs based on personal experience and preferences. There is significant variability in the duration of interruption between different neurosurgical practices. Moreover, thromboembolic complications have often been underestimated or not thoroughly reported in the literature.

Scores such as CHA₂DS₂-VASc or HAS-BLED estimate the risk of thromboembolic and bleeding events in patients with non-valvular atrial fibrillation [13], guiding therapeutic decisions. Considered risk factors include congestive heart failure (CHF), hypertension, age, diabetes mellitus, stroke, vascular disease, sex, abnormal liver and renal function, bleeding predisposition, labile INRs, and substance use. These scores have been proven to help predicting complications risk in the perioperative period of non-cardiac surgeries [4, 15, 20]. However, their role and application in the neurosurgical field have not yet been investigated.

The aim of this study is to evaluate the clinical outcomes and complications (such as acute rebleeding, recurrence, or thromboembolic events) up to 6 months in patients who underwent CSDH evacuation. We will compare patients who were taking anticoagulants or antithrombotic medications with a control group. Additionally, we will evaluate the role of CHA₂DS₂-VASc and HAS-BLED scores in predicting thromboembolic vascular events or hemorrhages.

## Materials and methods

This is a multicenter, prospective observational study with a case–control design, conducted in accordance with the STROBE [6] checklist for observational studies. The study was conducted in accordance with the World Medical Association Declaration of Helsinki and approved by the Local Ethical Committee (Internal Code: EM344-2023_1102/2020/Oss/AOUFe_EM1). We consecutively enrolled patients > 18 years old who were treated for CSDH surgical evacuation between 2021 and 2024. The exclusion criterion was a history of previous surgery for CSDH evacuation. The follow-up period was 6 months post-surgery. The follow-up procedure included immediate and 1-month post-operative CT scans, as well as complete clinical evaluations at 1 and 6 months post-operatively.

The co-primary endpoints of the study were the evaluation of:

- Hemorrhagic adverse events defined as: acute rebleeding of the previously operated CSDH, formation of contralateral CSDH, intracerebral hemorrhage, and clinically evident bleeding from the upper airways or gastrointestinal system.
- Thromboembolic adverse events defined as: radiologically evident ischemic stroke, myocardial infarction, upper or lower limb venous or arterial thrombosis, and pulmonary embolism.
- GOS at 1 and 6 months.

The secondary endpoints were the estimation of a statistical correlation between pre-surgical scores (CHA₂DS₂-VASc, HAS-BLED, Charlson Comorbidity Index, mRS, GCS) and the primary endpoints.

Several clinical and radiological variables were collected, including: hematoma characteristics, surgical procedure details, pharmacological therapy (including suspension and resumption time), post-operative complications, intraoperative need for pro-coagulant drugs, pre and post-operative blood examinations, functional follow up (Glasgow Outcome Scale, GOS) at 1 and 6 months, CHA2DS2-VASc, HAS-BLED, Charlson Comorbidity Index (CCI), modified Ranking Scale (mRS), Glasgow Coma Scale (GCS).

### Statistical analysis

Potential differences in the distribution of all recorded clinical characteristics and outcomes between subjects undergoing vs. those not undergoing anticoagulant-anti-PLT therapy were initially assessed using standard univariate analyses: chi-squared test for categorical variables, t-test and Kruskal–Wallis test for normally distributed and not normally distributed continuous variables, respectively (distribution was assessed through the Shapiro–Wilk test).

For each of the three primary outcomes (a. 1-month GOS between 4 and 6; (b) 6-month GOS between 4 and 6; (c) postoperative mRS between 1 and 3), the potential independent predictors were then separately evaluated using logistic regression. To reduce overfitting, all models were built including only the variables that were clinically relevant, with the exception of age, emergency surgery, CHA2DS2-VASc score and HAS-BLED score, that were included a priori. In all models, the two mentioned scores and the CCI were treated both as continuous and ordinal, categorical variables, identifying three categories for CHA2DS2-VASc and HAS-BLED, and two categories for CCI (< 5 vs. ≥ 5).

Standard diagnostic procedures were adopted to validate all models, including: Influential observation analysis (Dbeta, change in Pearson chi-square); Hosmer–Lemeshow test for goodness of fit; C statistic (area under the Receiver Operating Characteristic [ROC] curve).

Statistical significance was defined as a two-sided *p*-value < 0.05. All analyses were carried out using Stata, version 13.1 (Stata Corp., College Station, TX, 2014).

## Results

Table 1 reports the demographic, clinical, and radiological characteristics of the patients. A total of 340 patients were enrolled in this study, with 221 patients in the anticoagulant-antiplatelet therapy (AAPT) group and 119 in the no-therapy (NT) group.

**Table 1:** Demographic and clinical characteristics of the sample of patients with chronic subdural hematoma, overall and by therapy status (anticoagulant/antiplatelet—AAPT versus no therapy—NT)

	Overall sample	AAPT	NT	
Variables	(*N*=340)	(*N*=221)	(*N*=119)	*p* *
Mean age (SD)	78.1 (9.9)	80.6 (7.8)	73.5 (11.5)	<0.001
Male gender, %	36.5	38.9	31.9	0.2
*CSDH characteristics:*				
Bilateral hematoma	30.0	31.7	26.9	0.4
Mean thickness in mm (SD)	22.0 (6.5)	21.8 (6.8)	22.4 (5.8)	0.4
Acute/subacute components, %	40.1	39.1	40.0	0.6
*Emergency surgery:*				
Burr hole, %	(*N*=201)	(*N*=111)	(*N*=90)	
- Single	19.9	19.8	20.0	0.9
- Double	9.0	9.9	7.8	0.8
	(N=220)	(N=122)	(N=98)	
Minicraniectomy, %	13.6	18.9	7.1	0.012
Opening of the visceral membrane, %	12.3	13.1	10.8	0.6
*Delayed surgery:*				
Burr hole, %	(*N*=277)	(*N*=181)	(*N*=96)	
- Single	44.4	53.6	27.1	<0.001
- Double	11.2	12.7	8.3	0.3
	(*N*=215)	(*N*=119)	(*N*=96)	
Minicraniectomy, %	47.0	40.3	55.2	0.030
Opening of the visceral membrane, %	56.9	65.0	52.6	0.071
Subdural drainage, %				
- None	28.5	19.9	44.5	<0.001
- Anterior-posterior	55.6	62.0	43.7	0.001
- Posterior-anterior	15.9	18.1	11.8	0.13
Charlson Comorbidity Index:				
Mean score (SD)	4.5 (1.9)	3.4 (1.7)	5.1 (1.7)	<0.001
Score categories, %:				<0.001
- <5	53.2	38.5	80.7	
- >=5	46.8	61.5	19.3	

* Chi-squared test for categorical variables; t-test and Kruskal-Wallis test for parametric and non-parametric continuous variables, respectively. *SD* standard deviation

The mean age for the overall sample was 78.1 years (SD = 9.9), with the AAPT group averaging 80.6 years (SD = 7.8) and the NT group averaging 73.5 years (SD = 11.5) (*p* < 0.001). Males comprised 36.5% of the patients, with 38.9% in the AAPT group and 31.9% in the NT group (p = 0.2).

### CSDH radiological characteristics

Bilateral hematoma was present in 30.0% of the overall population, 31.7% in the AAPT group and 26.9% in the NT group (*p* = 0.4—Table 1).

### Surgery

Emergency surgery was performed in 87 patients (25.6%). There was no statistically significant difference between the groups regarding the surgical technique used (single/double burr hole vs. minicraniectomy; *p* = 0.9). Minicraniectomy (29 patients) was more frequently performed in the AAPT group (18.9% vs. 7.1%, *p* = 0.012).

Delayed surgery (more than 5 days after admission) was performed in 253 patients (74.4%). A single burr hole was preferred in the AAPT group compared to the NT group (53.6% vs. 27.1%, *p* < 0.001), while minicraniectomy was more common in the NT group (55.2% vs. 40.3%, *p* = 0.03).

Overall, subdural drainage was placed in 243 (71.5%) patients, with a preference for an anterior–posterior direction (*p* = 0.001). In the NT group, 44.5% of patients had no drainage, compared to 19.9% in the AAPT group. This difference was statistically significant (*p* < 0.0001, see Table 1).

### Charlson Comorbidity Index (CCI)

The mean CCI score was 4.5 in the overall population, 5.1 in the AAPT group, and 3.4 in the NT group (*p* < 0.001). Most patients in the NT group (80.7% vs. 38.5% in the AAPT group, *p* < 0.001) had a score < 5 (see Table 1).

### Risk factors and comorbidities for thromboembolic risk

Among the most important risk factors, smoking, diabetes, hypertension, acute myocardial infarction (AMI), atrial fibrillation (AF), heart valve presence, CHF, history of stroke/TIA or thromboembolism, and peripheral vascular disease were all more commonly present in the AAPT group, with significant statistical differences (see Table 2).

**Table 2:** Risk factors and comorbidities for *thromboembolic risk* of the sample of patients with chronic subdural hematoma, overall and by therapy status (anticoagulant/antiplatelet—AAPT versus no therapy—NT)

	Overall sample	AAPT	NT	
Variables	(*N*=340)	(*N*=221)	(*N*=119)	*p* *
Current or past smoke, %	18.5	23.8	7.9	0.001
Alcohol abuse, %	4.5	3.6	6.2	0.3
Diabetes, %	23.6	30.8	9.3	<0.001
Hypertension, %	72.9	83.4	52.7	<0.001
Acute myocardial infarction, %	22.3	32.2	2.0	<0.001
Atrial fibrillation, %	21.0	30.3	2.1	<0.001
Heart valve, %	7.5	10.6	1.0	0.003
Congestive heart failure, %	9.8	14.2	1.0	<0.001
Stroke/TIA or thromboembolism, %	17.0	22.8	5.1	<0.001
Vascular disease ^A^, %	25.2	36.7	2.0	<0.001
CHA2DS2-VASc score:				
Mean score (SD)	3.7 (1.7)	4.4 (1.5)	2.4 (1.4)	<0.001
Score categories, %:				
- 0–2	25.6	10.0	54.6	<0.001
- 3–5	59.7	68.3	43.7	<0.001
- 6–8	14.7	21.7	1.7	<0.001

* Chi-squared test for categorical variables; t-test and Kruskal-Wallis test for parametric and non-parametric continuous variables, respectively. *SD* standard deviation.  ^A^ Peripheral artery disease or aortic plaque

The CHA₂DS₂-VASc mean score was 3.7 in the overall population, 4.4 in the AAPT group, and 2.4 in the NT group, showing a statistically significant difference (*p* < 0.001).

The low-risk category (0–2 points) was significantly more represented in the NT group compared with the AAPT group (54.6% vs. 10.0%; *p* < 0.001). Moderate and high-risk categories (3–5 and 6–8 points) were much more frequently reported in the AAPT group compared with the NT group (68.3% vs. 42.7%, *p* < 0.001; and 21.7% vs. 1.7%, *p* < 0.001, respectively).

Further details are provided in Table 2..

### Risk factors and comorbidities for hemorrhagic risk

Among the factors influencing hemorrhagic risk, a labile INR (unstable or elevated INRs, with time in therapeutic range < 60%) was significantly more prevalent in the AAPT group compared to the NT group (6.5% vs. 0.0%, *p* = 0.010). Other risk factors such as kidney or liver disease, or a history of major bleeding, did not show statistically significant differences between the two groups (see Table 3).

**Table 3:** Risk factors and comorbidities for* hemorrhagic risk* of the sample of patients with chronic subdural hematoma, overall and by therapy status (anticoagulant/antiplatelet - AAPT versus no therapy - NT)

	Overall sample	AAPT	NT	
Variables	(*N*=340)	(*N*=221)	(*N*=119)	*p* *
Kidney disease, % ^B^	7.9	9.2	5.1	0.2
Liver disease, % ^C^	4.1	3.6	5.2	0.5
Major bleeding or predisposition, %	2.1	1.0	4.1	0.08
Labile INR, % ^D^	4.4	6.5	0.0	0.010
HAS-BLED score:				
Mean score (SD)	2.5 (1.2)	3.1 (0.8)	1.5 (1.0)	<0.001
Score categories, %:				
- 0–2	45.3	22.6	87.4	<0.001
- 3–4	51.5	72.4	12.6	<0.001
- 5–9	3.2	5.0	0.0	0.015

* Chi-squared test for categorical variables; t-test and Kruskal-Wallis test for parametric and non-parametric continuous variables, respectively. *SD* standard deviation.  ^B^ Dialysis, transplant, Cr >2.26 mg/dL or >200 µmol/L.  ^C^ Cirrhosis or bilirubin >2x normal with AST/ALT/AP >3x normal. ^D^ Unstable/high INRs, time in therapeutic range <60%

The mean HAS-BLED score for the entire study population was 2.5, with a notable disparity between the AAPT and NT groups (3.1 vs. 1.5, respectively; *p* < 0.001). The low-risk category (0–2 points) was predominant in the NT group compared to the AAPT group (87.4% vs. 22.6%, *p* < 0.001). In contrast, moderate (3–4 points) and high-risk categories (5–9 points) were more frequently observed in the AAPT group (72.4% and 5.0%, respectively), with statistically significant differences compared to the NT group (12.6%, *p* < 0.001 and 0.0%, *p* = 0.015).

### Pre and post-surgical laboratory parameters

The median pre-surgical INR for the entire population was 1.09. Specifically, in the AAPT group, it was 1.10, and in the NT group, it was 1.06, showing a statistically significant difference (*p* = 0.001). Pre-surgical platelet count, median aPTT, and PT did not exhibit significant differences between the two groups.

Similarly, for post-surgical INR, the median value for the entire cohort was 1.10. In the AAPT and NT groups, the median INR was 1.10 and 1.05, respectively, with a statistically significant difference noted (*p* = 0.008). Post-surgical platelet count, aPTT, and PT did not differ significantly between the groups (see Table 4).

**Table 4:** Laboratory exams and transfusion of the sample of patients with chronic subdural hematoma, overall and by therapy status (anticoagulant/antiplatelet—AAPT versus no therapy—NT)

	Overall sample	AAPT	NT	
Variables	(*N* = 340)	(*N* = 221)	(*N* = 119)	*p* *
Pre-surgical laboratory parameters				
Platelets, mean count/µL (SD)	225 (81.9)	221 (86.4)	233 (72.4)	0.2
INR, median (IQR)	1.09 (1.01–1.20)	1.10 (1.02–1.20)	1.06 (1.01–1.11)	0.001
aPTT, median (IQR)	1.05 (0.92–26.6)	1.02 (0.92–25.7)	1.08 (0.92–29.0)	0.5
PT, median (IQR)	1.10 (1.03–1.97)	1.12 (1.03–2.27)	1.10 (1.03–1.26)	0.12
Post-surgical laboratory parameters				
Platelets count/µL, mean (SD)	220 (72.5)	214 (72.2)	232 (72.0)	0.03
INR, median (IQR)	1.10 (1.01–1.17)	1.10 (1.02–1.20)	1.05 (1.00–1.13)	0.008
aPTT, median (IQR)	1.00 (0.91–25.5)	1.00 (0.90–1.18)	1.07 (0.92–29.0)	0.6
PT, median (IQR)	1.10 (1.03–1.40)	1.10 (1.04–1.43)	1.10 (1.01–1.30)	0.2
Peri/intraoperative antagonists transfusion				
	(*N* = 293)	(*N* = 196)	(*N* = 97)	
Platelets, %	7.9	11.2	1.0	0.002
FFP, %	3.1	4.7	0.0	0.03
Vitamin K, %	8.6	13.0	0.0	< 0.001

^*^ Chi-squared test for categorical variables; t-test and Kruskal–Wallis test for parametric and non-parametric continuous variables, respectively. *SD* Standard deviation

### Peri/intraoperative antagonist transfusion

Overall, platelet transfusion was necessary for 7.9% of patients, with significantly higher rates observed in the AAPT group (11.2%) compared to the NT group (1.0%) (*p* = 0.002 – see Table 4).

Additionally, fresh frozen plasma (FFP) and vitamin K were exclusively administered to patients in the AAPT group, with utilization rates of 4.7% and 13.0%, respectively.

### Pre-operative clinical conditions and post-operative outcome

The mean pre-operative mRS score for the entire cohort was 2.9, with specific scores of 3.2 in the AAPT group and 2.4 in the NT group, demonstrating a significant difference (*p* < 0.001 – see Table 5).

**Table 5:** Pre-operative clinical conditions and post-operative outcome in the sample of patients with chronic subdural hematoma, overall and by therapy status (anticoagulant/antiplatelet—AAPT versus no therapy—NT)

	Overall sample	AAPT	NT	
Variables	(*N* = 340)	(*N* = 221)	(*N* = 119)	*p* *
Modified Rankin Scale (mRS)				
- Mean preoperative score (SD)	2.9 (1.5)	3.2 (1.4)	2.4 (1.5)	< 0.001
- Mean postoperative score (SD)	2.0 (1.6)	2.4 (1.5)	1.3 (1.5)	< 0.001
- Mean pre-postoperative variation (SD)	0.9 (1.2)	0.8 (1.1)	1.1 (1.3)	0.06
Paired t-test p**	< *0.001*	< *0.001*	< *0.001*	
Post-operative mRS categories, %				
- 0–2	62.0	52.3	79.8	< 0.001
- 3	17.1	22.7	6.7	< 0.001
- 4–6	20.9	25.0	13.5	0.013
Glasgow Coma Scale (GCS)				
- Mean preoperative score (SD)	13.8 (1.8)	13.7 (2.0)	14.1 (1.4)	0.06
- Mean postoperative score (SD)	14.5 (1.6)	14.4 (1.7)	14.6 (1.4)	0.03
- Mean pre-postoperative variation (SD)	−0.6 (1.5)	−0.7 (1.7)	−0.5 (1.0)	0.2
Paired t-test p**	< *0.001*	< *0.001*	< *0.001*	
Post-operative GCS categories, %				0.9
- 3–9	2.7	2.7	2.5	
- 10–12	3.5	3.6	3.4	
- 13–15	93.8	93.7	94.1	
Post-operative CT scan:				
Haematoma volume reduction > 70%, %	75.5	75.6	75.5	0.9
Massive pneumocephalus, %	2.5	1.7	3.9	0.2
Follow-up				
Glasgow Outcome Scale (GOS):				
One-month mean score (SD)	4.2 (1.0)	4.1 (1.1)	4.5 (0.9)	0.001
Six-month mean score (SD)	4.3 (1.2)	4.1 (1.2)	4.6 (1.0)	0.001
Paired t-test p**	*0.9*	*0.5*	*0.9*	
One-month GOS categories, %				0.003
- 1–3	22.4	27.4	12.9	
- 4–6	77.6	72.6	87.1	
Six-month GOS categories, %				0.048
- 1–3	17.0	20.0	11.4	
- 4–6	83.0	80.0	88.6	

^*^ Chi-squared test for categorical variables; t-test and Kruskal–Wallis test for parametric and non-parametric continuous variables, respectively. ** *P*-value of the comparison between pre- and post-operative scales. *SD* Standard deviation; *IQR* Interquartile range

^A^ Stroke, acute myocardial infarction or other events. ^B^ Occurring after surgery. ^C^ Seizures (*n* = 15); infections (*n* = 4); contusions (*n* = 2); controlateral hemorrhage (*n* = 1)

Mean post-operative mRS score was 2.0 for the entire population, with scores of 2.4 in the AAPT group and 1.3 in the NT group, indicating a significant difference (*p* < 0.001).

Post-operatively, the most frequent mRS category (indicating favorable outcome, 0–2) was observed in 62.0% of the overall population, with rates of 52.3% and 79.8% in the AAPT and NT groups, respectively (*p* < 0.001 – see Table 5).

Post-operative CT scans revealed a reduction in hematoma volume in 75.5% of patients, with no statistically significant differences observed between the two groups.

The mean GOS score at one month was 4.2 for the entire cohort, specifically 4.1 in the AAPT group and 4.5 in the NT group, demonstrating a significant difference (*p* < 0.001). Similar results were observed for the six-month GOS scores (see Table 5).

Most patients (77.6%) achieved a favorable outcome (GOS 4–6) at one month, with rates of 72.6% in the AAPT group and 87.1% in the NT group (*p* = 0.003). Similar results were observed for the six-month GOS category (see Table 5).

### Complications

Acute post-operative hemorrhages occurred in 16 patients (5.5% of the total cohort), with 15 cases in the AAPT group and 1 case in the NT group, showing a significant difference (*p* = 0.02 – see Table 6). The median time from surgery to acute hemorrhage was 5 days.

**Table 6:** Complications in the sample of patients with chronic subdural hematoma, overall and by therapy status (anticoagulant/antiplatelet—AAPT versus no therapy—NT)

	Overall sample	AAPT	NT	
Variables	(*N* = 340)	(*N* = 221)	(*N* = 119)	*p* *
	(*N* = 290)	(*N* = 193)	(*N* = 97)	
Post-operative hemorrhage, % (n)	5.5 (16)	7.8 (15)	1.0 (1)	0.02
Median time between surgery and hemorrhage, days (IQR)	5.0 (1.0–7.5)	5.0 (1.0–8.0)	–	–
Median time between drug resumption and hemorrhagic events, days (IQR)	30.5 (1.0–60.0)	30.5 (1.0–60.0)	–	–
Post-operative thromboembolic events, % (n) ^A^	7.9 (27)	10.0 (22)	4.2 (5)	0.06
Median time between drug discontinuation and thromboembolic events, days (IQR)	7.0 (3.0–38.0)	7.0 (4.0–27.0)	5.0 (2.0–45.0)	0.7
Recurrences, % ^B^	11.2 (38)	11.8 (26)	10.1 (12)	0.6
Median time between surgery and recurrences, days (IQR)	24.0 (9.0–310)	30.0 (10.0–60.0)	16.0 (7.0–24.0)	0.04
Other complications occurring during the follow-up, % ^C^	6.2 (21)	5.9 (13)	6.7 (8)	0.9

^*^ Chi-squared test for categorical variables; t-test and Kruskal–Wallis test for parametric and non-parametric continuous variables, respectively. ** *P*-value of the comparison between pre- and post-operative scales. *SD* Standard deviation. *IQR* Interquartile range

^A^ Stroke, acute myocardial infarction or other events. ^B^ At least 7 days after surgery. ^C^ Seizures (*n* = 15); infections (*n* = 4); contusions (*n* = 2); controlateral hemorrhage (*n* = 1)

Among the 15 AAPT patients experiencing post-operative hemorrhage, the drug was discontinued in 12 cases, with a median cessation time of 4.3 days.

Post-operative thromboembolic events were observed in 27 patients (7.9% of the total cohort), with 22 cases in the AAPT group and 5 in the NT group (*p* = 0.06). Specific events included pulmonary embolism (PE) and deep vein thrombosis (DVT) in 15 patients (12 in the AAPT group, 3 in the NT group), AMI in 6 patients (5 in the AAPT group, 1 in the NT group), and stroke in 6 patients (5 in the AAPT group, 1 in the NT group). Among the 22 AAPT patients with thromboembolic events, the median time from drug discontinuation to occurrence was 7 days.

Overall, recurrences were reported in 38 patients (11.2% of the total cohort), with 30 cases (11.8%) in the AAPT group and 8 cases (10.1%) in the NT group, showing no significant difference (*p* = 0.6) (see Table 6).

Among the 26 patients in the AAPT group who experienced recurrences, 20 had discontinued the drug before surgery, with a median cessation period of 4.6 days. In 6 of these cases, the drug was not discontinued.

The median time from surgery to recurrences was 24 days for the entire cohort, specifically 30 days in the AAPT group and 16 days in the NT group, indicating a statistically significant difference (*p* = 0.04).

Other complications occurred in 6.2% of all patients, with no statistically significant differences observed between the AAPT and NT groups (see Table 6).

In the AAPT group, the median time to resume the drug was 40 days.

### Multivariate analyses

Logistic regression models were performed to assess potential independent predictors of each primary favorable outcome (1-month GOS between 4 and 6 and 6-month GOS between 4 and 6) (see Table 7).

**Table 7:** Multivariate analyses evaluating the association between anticoagulant-anti-PLT therapy status and (a) 1-month Glasgow Outcome Scale (GOS1) between 4 and 6; (b) 6-month GOS (GOS6) between 4 and 6; (c) postoperative Modified Rankin Scale (post-op mRS) between 1and 3, adjusting for selected potential confounders

	GOS1 4–6 (*n* = 292)			GOS6 4–6 (*n* = 286)		
	%	Adj. OR (95% CI)	p	%	Adj. OR (95% CI)	*p*
Anticoagulant-anti-PLT therapy						
- No	87.1	1 (ref. cat.)	–	88.6	1 (ref. cat.)	–
- Yes	72.6	1.57 (0.60–4.14)	0.4	80.0	1.84 (0.63–5.42)	0.3
Age						
5-year increase	–	0.78 (0.61–0.98)	0.04	–	0.64 (0.49–0.85)	0.002
- ≤ 75 years	90.2	1 (ref. cat.)	–	94.6	1 (ref. cat.)	–
- 76–83 years	79.8	1.21 (0.47–3.11)	0.7	85.9	0.82 (0.26–2.59)	0.7
- ≥ 84 years	63.2	0.71 (0.28–1.75)	0.5	68.5	0.32 (0.11–0.96)	0.042
Emergency surgery						
- No	77.2	1 (ref. cat.)	–	82.7	1 (ref. cat.)	–
- Yes	74.3	1.65 (0.61–4.45)	0.3	82.9	2.08 (0.69–6.20)	0.2
Pre-operative INR, 1-point increase	–	1.10 (0.50–2.41)	0.8	–	1.17 (0.49–2.80)	0.7
Pre-operative mRS, 1-point increase	–	0.48 (0.36–0.64)	< 0.001	–	0.49 (0.35–0.67)	< 0.001
CHA2DS2-VASc score						
- 1-point increase	–	0.73 (0.55–0.97)	0.033	–	0.84 (0.62–1.16)	0.3
- 0–2	92.9	1 (ref. cat.)	–	94.1	1 (ref. cat.)	–
- 3–5	76.7	0.58 (0.17–1.95)	0.4	81.8	0.64 (0.17–2.44)	0.5
- 6–8	54.2	0.41 (0.09–1.80)	0.2	67.4	0.40 (0.08–2.04)	0.3
HAS-BLED score						
1-point increase	–	0.96 (0.61–1.50)	0.8	–	0.99 (0.61–1.61)	0.9
- 0–2	84.8	1 (ref. cat.)	–	85.9	1 (ref. cat.)	–
- 3–4	73.4	0.88 (0.36–2.14)	0.8	81.7	1.17 (0.45–3.02)	0.8
- 5–9	45.5	0.26 (0.04–1.52)	0.13	63.6	0.52 (0.09–3.16)	0.5
Charlson Comorbidity Index						
- 1-point increase	–	0.87 (0.70–1.08)	0.2	–	0.89 (0.71–1.12)	0.3
- < 5	86.1	1 (ref. cat.)	–	86.7	1 (ref. cat.)	–
- ≥ 5	67.7	0.80 (0.39–1.65)	0.5	78.5	1.59 (0.71–3.60)	0.3

*Adj.* Adjusted, *OR* Odds ratio, *CI* Confidence interval, *ref. cat.* reference category

The raw percentages refer to the proportion of subjects experiencing each outcome in each category of exposed vs. unexposed patients (e.g. proportion of subjects with 1-month GOS between 4 and 6 among subjects not using vs using anticoagulant-anti-PLT therapy)

For each outcome, two separate models were fit: the first including CHA2DS2-VASc, HAS-BLED and the Charlson Comorbidity Index as continuous variables, the second including the same three scores categorized into ordinal or categorical variables, with all other covariates remaining stable

Area under the ROC curve (AUC) model 1 = 0.81; AUC model 2 = 0.82; AUC model 3 = 0.90

Anticoagulant or antiplatelet therapy did not demonstrate a significant influence on 1-month or 6-month GOS outcomes.

Increasing age by 5 years was identified as a significant factor, associated with a lower probability of favorable outcomes for both 1-month (OR = 0.78, 95% CI 0.61–0.98, *p* = 0.04) and 6-month (OR = 0.64, 95% CI 0.49–0.85, *p* = 0.002) GOS scores.

Emergency surgery did not show a statistically significant influence on favorable outcomes.

A 1-point increase in pre-operative modified Rankin Scale (mRS) score was found to be a statistically significant factor (*p* < 0.001), associated with a lower probability of favorable outcomes at both 1 month (OR = 0.48, 95% CI 0.36–0.64) and 6 months (OR = 0.49, 95% CI 0.35–0.67) on the GOS scale.

Although CHA₂DS₂-VASc score categories did not reach statistical significance, a 1-point increase in the score was associated with a lower probability of favorable outcomes at 1 month (OR = 0.73, 95% CI 0.55–0.97, *p* < 0.033).

Increases in pre-operative INR by 1 point, HAS-BLED score, and CCI did not show a statistically significant influence on favorable outcomes.

### AAPT group analysis by time of drug discontinuation

An additional analysis was conducted on AAPT patients taking ASA (93 patients) with discontinuation before surgery > or ≤ 5 days (refer to Table 8).

**Table 8:** Selected clinical characteristics and outcomes among those patients under pharmacological treatment with ASA (N = 93), stratified by time of drug discontinuation before surgery (≤ 5 days vs. > 5 days)

	≤ 5 days	> 5 days	
Variables	(*N* = 59)	(*N* = 34)	*p* *
New bleeding, %	20.3	0.0	0.005
Recurrences, %	11.9	14.7	0.7
One-month GOS categories, %:			0.8
- 1–3	18.6	20.6	
- 4–6	81.4	79.4	
Six-month GOS categories, %:			0.7
- 1–3	15.5	12.1	
- 4–6	84.5	87.9	

^*^ Chi-squared test for categorical variables; t-test and Kruskal–Wallis test for parametric and non-parametric continuous variables, respectively

Statistically significant differences were observed, indicating a higher risk of new bleedings in the ASA group with discontinuation ≤ 5 days. However, no statistically significant differences were found in recurrences.

It’s important to note that this analysis was conducted on a relatively small number of patients, which may introduce bias.

## Discussion

### General considerations

This study showed that the use of anticoagulants or antiplatelets did not significantly affect the clinical outcomes of patients undergoing surgery for CSDH. Although initial analysis suggested better short- and long-term outcomes for patients not on these therapies, multivariate analysis did not confirm this.

Regarding surgery timing (emergency vs. delayed), no significant differences were noted between the groups. Emergency cases more often involved minicraniectomy in patients on anticoagulants, chosen for better bleeding control. In elective cases, minicraniectomy was preferred for improved hematoma exposure. Indeed, a study by Zolfaghari et al. [26] on 1003 patients suggested that despite burr-hole craniotomy and craniectomy have comparable outcomes in terms of recurrence rates and mortality, minicraniectomy was significantly associated with medical complications and serious surgical postoperative complications.

Multivariate analysis did not identify emergency surgery as a significant factor in improving outcomes. The optimal timing of surgery for CSDH remains debated. Early surgery may prevent neurological decline and reduce complications, but delays might be necessary to optimize patients with comorbidities.

While timely treatment is stressed, few studies focus on this in CSDH. Our findings did not show a clear benefit of emergency over delayed surgery on outcomes. Zolfaghari et al. were not able to identify any significant negative effect upon outcome correlated to time from diagnostic CT scan until surgery. On the other hand, Venturini et al. [21] concluded that the time of surgery is associated positively with the length of hospitalization, but not with the outcome, complications, recurrence, reoperation and survival. Further studies are needed to clarify surgery timing’s impact, but delaying necessary surgery is not advised based on our results.

### Bleeding risk and complications

Post-operative hemorrhages occurred in 5.5% of the overall study population, with a significant difference noted between groups (7.8% in the AAPT group vs. 1.0% in the NT group). Among the 15 AAPT patients with hemorrhages, treatment had been discontinued for an average of 4.3 days, and none required a second surgery for acute bleeding. These findings align with literature suggesting that preoperative AP or AC therapy may not markedly increase the risk of postoperative bleeding requiring surgical intervention in patients undergoing CSDH surgery [9, 10].

Recurrences were observed in 11.2% of all patients, with no statistically significant differences observed between the two groups.

The risk of recurrences in patients treated surgically for CSDH remains a topic of debate. A recent systematic review [3] suggested higher recurrence rates among patients on AC or AP (OR 1.41; CI = 1.11– 1.79; I2 = 28% and 1.31; CI = 1.08– 1.58; I2 = 32%). However, many studies included in the review did not initially report statistically significant odds ratios (OR), and the overall quality and heterogeneity of the evidence were acknowledged to be limitations. Furthermore, it was unclear how recurrence events correlated with antithrombotic reversal or medication washout, complicating the interpretation of these findings.

Contrarily, several recent clinical studies [12, 16, 23–25] have demonstrated no difference in recurrence risk between patients using AC or AP, including those who resumed these medications early after surgery. Consistent with our own experience and findings from previous studies [14, 18, 19], our data suggest that the use of these medications does not significantly influence the risk of CSDH recurrences.

Considering the interval between drug suspension and surgery is crucial. We conducted a subgroup analysis on AAPT patients taking ASA and dividing them based on half-life time.

Our findings suggest a potential difference in new bleeding incidents among patients operated on before versus after 5 days of ASA discontinuation, but no significant difference in recurrence rates was observed. Larger sample sizes are needed to explore this further.

The mean HAS-BLED score was 2.5 overall, higher in the AAPT group (3.1 vs. 1.5 in the NT group). Despite most NT group patients being low-risk (0–2 category), the majority of AAPT patients were moderate risk.

The higher risk category in the AAPT group primarily correlated with increased acute bleeding risk, but did not influence recurrence rates or overall clinical outcomes. Even among patients experiencing recurrences, the mean HAS-BLED score was 2.47, suggesting that general hemorrhage risk factors may not significantly impact recurrence rates.

In multivariate analysis (Table 3), a higher HAS-BLED score was not associated with worse clinical outcomes.

### Thromboembolic risk and complications

In addition to acute hemorrhages occurring in 5.5% of cases, our data indicated thromboembolic events in 7.9% of patients, with no significant differences between the two groups. These events occurred on average 7 days post-surgery.

Thromboembolic complications are infrequently discussed in literature, with bleeding risk typically receiving more attention while thromboembolic risks are often underestimated. Studies providing data on this risk are limited. [2, 11, 16, 22].

Given their older age, general comorbidities, and hospitalization for surgery, patients with CSDH should be considered at moderate to high risk for thromboembolic complications.

In the group experiencing thromboembolic complications, the mean CHA₂DS₂-VASc score was 5.1, with half of the patients scoring > 6 (high risk). Across the entire study population, 90% of patients in the AAPT group and 44% in the NT group were at moderate to high thromboembolic risk.

A meta-analysis by Brannigan et al. [3]. highlighted a significant finding: while antithrombotic therapy was associated with a 31–41% increased risk of recurrence in CSDH patients, it also showed a 274% increased risk of vasal-occlusive events.

Therefore, thromboembolic risk should be carefully considered in all CSDH patients, including those not taking medications. In cases involving concurrent atrial fibrillation or other cardiac conditions necessitating neurosurgical intervention, assessing the patient’s CHA₂DS₂-VASc score can offer valuable insights into their overall risk profile. For high-risk patients, early resumption of medications should be considered.

This study is the first to report data on the use of CHA₂DS₂-VASc and HAS-BLED scores in the CSDH population. Originally developed for assessing bleeding or thromboembolic risks in patients with atrial fibrillation receiving anticoagulation therapy, these scores were collected for all our patients regardless of atrial fibrillation or anticoagulation status.

We chose to include these scores because they are widely used in other surgical settings. Given the elderly population typically affected by CSDH, these scores could provide valuable preoperative risk assessment. They may help identify high-risk patients who could benefit from tailored perioperative interventions aimed at reducing adverse outcomes.

## Conclusions

Our study confirmed that the use of anticoagulants or antiplatelets does not appear to influence outcomes, complication rates, or recurrence in patients undergoing surgical treatment for CSDH. However, it is crucial to carefully assess hemorrhagic and thromboembolic risks. Validated scores such as CHA₂DS₂-VASc or HAS-BLED could aid in stratifying these risks, thereby enhancing the management of perioperative drug therapy.

## Data Availability

No datasets were generated or analysed during the current study.

## Declarations

### Data sharing

The data that support the findings of this study are available on request from the corresponding author. The data are not publicly available due to privacy or ethical restrictions.
